# Blood Parasites in Owls with Conservation Implications for the Spotted Owl (*Strix occidentalis*)

**DOI:** 10.1371/journal.pone.0002304

**Published:** 2008-05-28

**Authors:** Heather D. Ishak, John P. Dumbacher, Nancy L. Anderson, John J. Keane, Gediminas Valkiūnas, Susan M. Haig, Lisa A. Tell, Ravinder N. M. Sehgal

**Affiliations:** 1 Department of Biology, San Francisco State University, San Francisco, California, United States of America; 2 Department of Ornithology and Mammalogy, California Academy of Sciences, San Francisco, California, United States of America; 3 Lindsay Wildlife Museum, Walnut Creek, California, United States of America; 4 Sierra Nevada Research Center, USDA Forest Service Pacific Southwest Research Station, Davis, California, United States of America; 5 Institute of Ecology, Vilnius University, Vilnius, Lithuania; 6 USGS Forest and Rangeland Ecosystem Science Center, Corvallis, Oregon, United States of America; 7 Department of Medicine and Epidemiology, School of Veterinary Medicine, University of California Davis, Davis, California, United States of America; University of Liverpool, United Kingdom

## Abstract

The three subspecies of Spotted Owl (Northern, *Strix occidentalis caurina;* California, *S. o. occidentalis*; and Mexican, *S. o. lucida*) are all threatened by habitat loss and range expansion of the Barred Owl (*S. varia*). An unaddressed threat is whether Barred Owls could be a source of novel strains of disease such as avian malaria (*Plasmodium* spp.) or other blood parasites potentially harmful for Spotted Owls. Although Barred Owls commonly harbor *Plasmodium* infections, these parasites have not been documented in the Spotted Owl. We screened 111 Spotted Owls, 44 Barred Owls, and 387 owls of nine other species for haemosporidian parasites (*Leucocytozoon*, *Plasmodium*, and *Haemoproteus* spp.). California Spotted Owls had the greatest number of simultaneous multi-species infections (44%). Additionally, sequencing results revealed that the Northern and California Spotted Owl subspecies together had the highest number of *Leucocytozoon* parasite lineages (n = 17) and unique lineages (n = 12). This high level of sequence diversity is significant because only one *Leucocytozoon* species (*L. danilewskyi*) has been accepted as valid among all owls, suggesting that *L. danilewskyi* is a cryptic species. Furthermore, a *Plasmodium* parasite was documented in a Northern Spotted Owl for the first time. West Coast Barred Owls had a lower prevalence of infection (15%) when compared to sympatric Spotted Owls (*S. o. caurina* 52%, *S. o. occidentalis* 79%) and Barred Owls from the historic range (61%). Consequently, Barred Owls on the West Coast may have a competitive advantage over the potentially immune compromised Spotted Owls.

## Introduction

Emerging infectious diseases (EIDs) affecting wildlife appear to be increasing in number and have led to localized decreases in population sizes and species extinctions [1]–[4]. Wildlife diseases are especially relevant today due to the potential of zoonotics such as West Nile Virus, HIV, and H5N1 avian influenza [5]–[7]. EIDs are facilitated by the movement of vectors and pathogens due to environmental alterations caused by climate change and human impacts (e.g. deforestation or the introduction of invasive species) [8]. Diseases are also pertinent to conservation biology because endangered or threatened species may be pushed to extinction due to direct mortality or indirectly by a reduced reproductive success [9], [10].

The Spotted Owl (*Strix occidentalis*) is threatened not only by habitat loss, but also an invasive owl species. All three subspecies of the Spotted Owl (Northern, *S. o. caurina;* California, *S. o. occidentalis*; and Mexican, *S. o. lucida*) have been the focus of intense conservation efforts. This study focuses on the Northern and California Spotted Owls since they are the two subspecies currently most impacted by the Barred Owl (*Strix varia*) range expansion [11]–[15].

Over the past fifty years, Barred Owls have expanded their historic range in the eastern United States by crossing through the southwestern region of Canada and moving down the Pacific Coast of Canada and the U.S. [11]. Barred Owls began their range expansion in British Columbia in 1943, and by 2007, were a prime reason for near extirpation of Spotted Owls in the province [16], [17]. In the United States, Barred Owls were first recorded in Washington in 1965, in Oregon in 1974 [18], and in California in 1981 [16]. Presently, they continue to move south, increasing in population size throughout the Spotted Owl range [11], [19]. Barred Owls pose a threat to Spotted Owls because they are more aggressive than Spotted Owls and compete for food and nesting resources [11], [20]. Expansion of Barred Owls may have additional adverse effects on Spotted Owl populations if they introduce novel infectious diseases.

Over two decades of intense research on Spotted Owls has made them one of most studied birds in the world. However, little is known about their blood parasites or disease threat [15], [21]–[23]. In one of only two studies of blood parasites in Spotted Owls, Gutiérrez (1989) showed that every Spotted Owl tested had at least one blood parasite (*Leucocytozoon* or *Haemoproteus* spp.) and 79% had simultaneous multi-species infections [19, Table 1]. Interestingly, neither study by Greiner (n = 1 individual) or Gutiérrez (n = 105) recorded *Plasmodium* infections in the Spotted Owl [19], [22].

**Table 1:** Previous research on Spotted and Barred Owl haematozoa

Species	n	# infected	L	H	P	Citation
*Strix occidentalis*	1	1	1	1	0	24
*S. o. occidentalis*	76	76	71	67	0	21
*S. o. caurina*	22	22	21	11	0	21
*S. o. lucida*	7	7	4	3	0	21
**Total**	**106**	**106**	**97**	**82**	**0**	
*Strix varia*	54	3	n/a	n/a	3	28
*Strix varia*	28	19	0	19*	3	31
*Strix varia*	21	19	19	2	0	29
*Strix varia*	5	1	0	1	0	26
*Strix varia*	4	3	3	2	3	24
*Strix varia*	3	2	2	1	2	27**
*Strix varia*	1	1	1	1	1	30**
*Strix varia*	1	1	n/a	n/a	1	25
*Strix varia*	1	0	0	0	0	32
**Total**	**64**	**42**	**22**	**24**	**6**	

L = Leucocytozoon, H = Haemoproteus, P = Plasmodium

* This number does not account for multiple Haemoproteus species.

** Multiple infections were found.

At least nine studies have analyzed blood parasites in one or more Barred Owls (n = 64; Table 1) [24]–[32]. From these studies, 43 of 64 (67%) Barred Owls examined were infected with at least one blood parasite. There were seven documented cases of *Plasmodium* infections (Table 1) including discovery of a new taxonomically distinct *Plasmodium* species (subgenus *Novyella*) [25]. Telford et al. (1997), found Barred Owls had longer and wider asexually dividing cells (a.k.a. schizonts) of *P. forresteri* than seven other raptor species living in Florida, suggesting that they might have a morphologically distinct *Plasmodium* strain [28].

Nine morphologically distinct haemosporidian blood parasites (order Haemosporidia) have been recorded in owls: *Haemoproteus noctuae, H. syrnii*, *Plasmodium subpraecox*, *P. fallax*, *P. forresteri, P. gundersi*, *P. hexamerium*, *P. elongatum*, and one species of *Leucocytozoon, L. danilewskyi* ( = *L. ziemanni*) [33]. While the concept of a species is currently in flux, there is evidence of cryptic speciation of haemosporidian blood parasites from genetic sequencing [34]–[36]. There is also evidence for genetic variation in the cytochrome *b* gene within microscopically-defined morphospecies [37], [38]. *Plasmodium*, *Haemoproteus*, and *Leucocytozoon* spp. are spread to avian hosts via insect vectors, mosquitoes (Culicidae), biting midges (Ceratopogonidae), hippoboscid flies (Hippoboscidae), and black flies (Simuliidae), respectively [33]. In general, *Plasmodium* spp. are thought to be more pathogenic than *Haemoproteus* or *Leucocytozoon* because they display a lower degree of host specificity, and cause a more severe blood pathology [33], [39]–[41]. However, numerous species of *Leucocytozoon* and some hemoproteids also cause disease in birds [33], [42]–[44]; thus different species of haemosporidian parasites can have differing effects on avian hosts.

Blood parasites are indicators of immune quality in birds, and parasite prevalence data can be used to reveal information about individual and population fitness [45]–[48]. Many species of blood parasites are generally thought to be harmless because they appear in otherwise healthy looking birds [45]. However, research has shown that blood parasites can have negative fitness impacts on the host [47]–[53]. Parasites can be pathogenic during energy-demanding or stressful phases of a host's life such as the first year [49], [50], migration [51], [52], breeding [48], [53], [54], and years of low food abundance [54], [55]. Research has also shown that parasitic infections can negatively impact reproductive success by delaying arrival to the breeding grounds [56], reducing clutch sizes [47], [54], [57], reducing nest defense behavior [58], [59], increasing probability of clutch desertion [60], reducing hatching success [47], [60], reducing fledging success [47], and siring nestlings with poorer body condition [57]. From a global perspective, cumulative effects of blood parasites on individuals can have serious consequences on host populations [45]. Blood parasites can also be extremely virulent when introduced to an immunologically naïve species [4], [33], [43], [44].

We surveyed haemosporidian parasites from the blood of twelve western North American owl species using PCR and DNA sequencing techniques. We examined the phylogenetic relationships, host specificities, and distributions among hosts with the intention to determine whether Barred Owls may be the source of novel parasites to Spotted Owl populations on the West Coast of North America. We also studied blood samples from Europe and Africa to help elucidate phylogeographic relationships of haemosporidian parasites in owls.

## Results

### Prevalence of Blood Parasites

Five hundred forty two individuals (317 belonging to the Strigidae, and 225 belonging to the Tytonidae) from twelve owl species were tested for *Plasmodium*, *Haemoproteus*, and *Leucocytozoon* spp. parasites (Table 2) using PCR techniques. In the Strigidae family, the overall prevalence of infection was 62% (n = 197), while 24% (n = 54) of the Tytonidae family had at least one blood parasite infection. Prevalences for the three haemosporidian parasites varied within and between families.

**Table 2:** Prevalence of haematozoa infections in Strigiformes

Family	Scientific Name	Common Name	*Code	Location	n	I (%)	MI (%)	L (%)	H (%)	P (%)
Strigidae	*Aegolius funereus*	Boreal Owl	BOOW	Lithuania	5	4	2	4	0	2
	*Asio otus*	Long-earred Owl	LEOW	CA, Lithuania	28	23 (82)	10 (36)	22 (79)	10 (36)	1 (4)
	*Athene cunicularia*	Burrowing Owl	BUOW	CA	7	1	0	1	0	0
	*Bubo virginianus*	Great-horned Owl	GHOW	CA	54	34 (63)	4 (7)	34 (63)	6 (11)	3 (6)
	*Glaucidium sjostedti*	Sjostedti Owl	n/a	Cameroon	2	2	2	2	2	0
	*Megascops kennicottii*	Western Screech Owl	WESO	CA	53	45 (85)	10 (25)	42 (79)	0 (0)	15 (28)
	*Otus scops*	Scops Owl	n/a	Khazakstan	2	0	0	1	0	0
	*Strix aluco*	Tawny Owl	n/a	Lithuania	2	0	0	0	0	0
	*Strix o. caurina*	Spotted Owl, Northern	SPOWno	CA, OR, WA	63	33 (52)	10 (16)	25 (40)	16 (25)	1 (2)
	*Strix o. occidentalis*	Spotted Owl, California	SPOWca	CA	48	38 (79)	21 (44)	29 (60)	30 (63)	0 (0)
	*Strix varia* **	Barred Owl	BDOW	MN, WI, TX	18	11 (61)	2 (11)	1 (6)	6 (33)	6 (33)
	*Strix varia* **	Barred Owl	BDOW	CA, OR, WA	26	4 (15)	1 (4)	2 (8)	1 (4)	2 (8)
	*S. o.* and *S. v.* hybrid	Spotted/Barred hybrid	n/a	OR	8	1	0	2	0	0
	*Strix woodfordii*	African Wood Owl	n/a	Cameroon	1	1	1	1	1	0
	**TOTAL**				**317**	**197 (62)**	**66 (21)**	**166 (52)**	**72 (23)**	**30 (9)**
Tytonidae	*Tyto alba pratincola*	Barn Owl	BNOW	CA	180	54 (30)	3 (2)	54 (30)	3 (2)	0 (0)
	*Tyto alba guttata*	German Barn Owl	n/a	Denmark	45	0 (0)	0 (0)	0 (0)	0 (0)	0 (0)
	**TOTAL**				**225**	**54 (24)**	**3 (1)**	**54 (24)**	**3 (1)**	**0 (0)**

I = Infected, MI = multiple infections, L = *Leucocytozoon*, H = *Haemoproteus*, P = *Plasmodium*

* American Ornithologist's Union (AOU) name code

** *Strix varia* was split into two groups to due differing prevalences on the West Coast vs their historic range.

PCR and microscopy techniques indicated blood parasite prevalences ranged from zero in Barn Owls from Denmark (n = 45), to 100% in the African owls. The African owls (n = 3) had at least three blood parasite species identified by blood smear analysis. One individual Sjöstedt's Owlet (*Glaucidium sjostedti*) was infected with five morphologically distinct haemosporidians (*Leucocytozoon danilewskyi*, *Haemoproteus noctuae*, *Haemoproteus syrnii*, *Plasmodium* (subgenus *Haemamoeba*) sp., and *Plasmodium* (subgenus *Giovannolaia*) sp.).

Prevalences also varied between Spotted Owl subspecies in this study. The California Spotted Owl was significantly more likely to be infected with a blood parasites (p<0.007; χ^2^ = 7.36) and also has significantly more multiple infections (p = 0.002; χ^2^ = 9.18) than the Northern Spotted Owl. When compared to owls belonging to the same taxonomic family (Strigidae total numbers from Table 2), the California Spotted Owl and Northern Spotted Owls had significantly more *Haemoproteus* infections (*S. o. occidentalis* p = 0.0001, χ^2^ = 30.83; *S. o. caurina* p = 0.0001, χ^2^ = 15.36), and the California Spotted Owl had significantly less *Plasmodium* infections (p = 0.05; χ^2^ = 3.77). Even though the Northern Spotted Owl prevalence was also low (n = 1), it was not significantly different from the other Strigidae owls. However, finding one individual infected with a *Plasmodium* infection is significant because it is the first documentation of Spotted Owls with this parasite.

Northern Spotted Owl samples were further tested for change in blood parasite prevalence between ten-year time spans (1994–1996 to 2004–2005) but no statistical differences were found (Table 3). Furthermore, blood parasite prevalences did not differ within the Northern Spotted Owls when compared between states (WA vs. OR) (Table 3).

**Table 3:** Prevalence of blood parasites in Northern Spotted Owls collected over ten years

Year Collected	Location	n	# infected (%)	L (%)	H (%)
1994–1996	Washington	16	7 (43.8)	7 (43.8)	2 (12.5)
1994–1996	Oregon	6	3 (50)	3 (50)	1 (16.7)
	**Total**	**22**	**10 (45.5)**	**10 (45.5)**	**3 (13.6)**
2005	Washington	7	3 (42.8)	2 (28.6)	1 (14.3)
2004–2005	Oregon	21	13 (61.9)	6 (28.6)	8 (38.1)
	**Total**	**28**	**16 (57)**	**8 (28.6)**	**9 (32.1)**

L = *Leucocytozoon*, H = *Haemoproteus*

Barred Owl results were divided into two subgroups to examine potential geographic differences (CA, OR, WA vs MN, WI, TX; Table 2). Barred Owls in the historic eastern range had significantly higher prevalences (p = 0.001; χ^2^ = 12.36) and significantly more *Plasmodium* infections (p = 0.05; χ^2^ = 4.70) than the Barred Owls on the west coast.

The blood parasite prevalence also differed between western Barred Owls and Spotted Owls. Spotted Owls had a higher prevalence of blood parasites (52% in Northern subspecies, 79% in California subspecies) than Barred Owls on the West Coast (15%). These sympatric Barred Owls had significantly lower prevalences of *Leucocytozoon* (p = 0.001; χ^2^ = 28.75) and *Haemoproteus* infections (p = 0.001; χ^2^ = 25.80), and fewer cases of multiple infections (p = 0.001; χ^2^ = 20.56) when compared to all Spotted Owls. Barred Owls, however, did have significantly higher *Plasmodium* spp. prevalences (p = 0.05; χ^2^ = 4.17), but the differences in *Plasmodium* prevalence were more pronounced when comparing all Spotted Owls with all Barred Owls sampled (p = 0.001; χ^2^ = 14.38) (see Table 2).

### DNA Sequencing and Host-Parasite Relationships

#### Leucocytozoon

**Figure 1:**
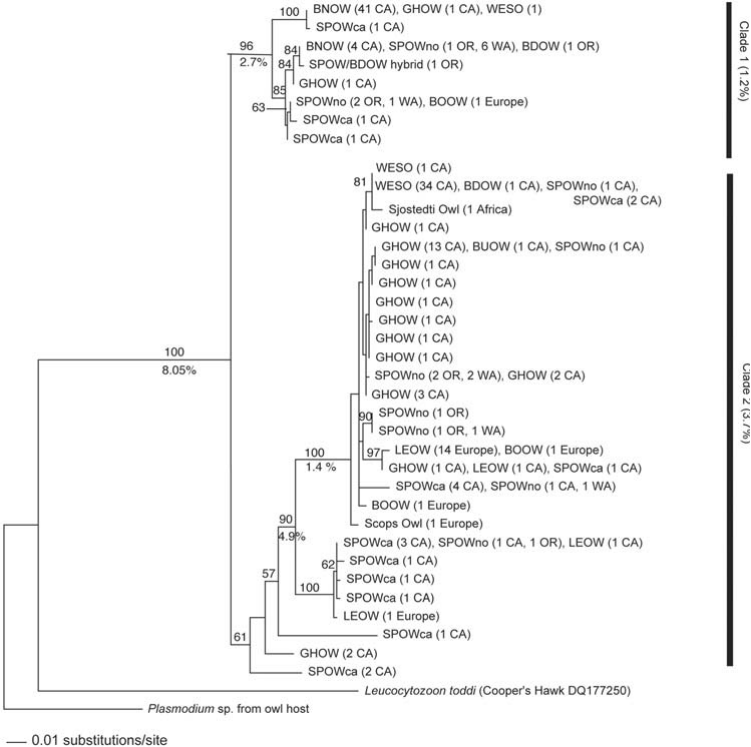
Maximum likelihood tree (1) of *Leucocytozoon* from eleven owl species. Maximum likelihood bootstrap values are shown above the branches (100 replicates) and uncorrected ‘p’ distances are shown below. For explanation of bird abbreviation codes, see Appendix 1. Following each bird abbreviation code is the number of individuals (n) with that particular lineage and the sampling location.

The *Leucocytozoon* dataset consisted of 623 base pairs of the mitochondrial cytochrome *b* gene sequenced from 181 owls among eleven species. There were 38 lineages found (including two outgroups) with 219 variable characters and 124 parsimony informative characters. The maximum likelihood heuristic search for the *Leucocytozoon* analysis yielded 34 trees which varied only at the branch tips (Figure 1).

The likelihood tree revealed two *Leucocytozoon* clades (1 & 2) with relatively high sequence divergence (uncorrected *p* = 8.05%). The presence of two distinct clades suggests that *L. danilewskyi* is a cryptic species. A side by side blood smear analysis of *Leucocytozoon* from blood parasites within Boreal Owls from both clades showed identical morphologies. However, the Barn Owls, which were only found in the first clade, only had the round morph gametocytes of *Leucocytozoon* present in the blood smears. Typically, elongated and round morph gametocytes develop in *Leucocytozoon danilewskyi* infections, and they are frequently present simultaneously. In summary, although the morphology of *L. danilewskyi* appears identical in all blood smears, the mtDNA reveals that there are many different haplotypes/lineages and high genetic divergence, perhaps justifying reclassifying *L. danilewskyi* into two or more species or subspecies.

The Strigidae family appears to be susceptible to parasites from both clades of *Leucocytozoon* spp (clade 1 & 2, Figure 1). The Tytonidae (Barn Owls) only had parasite strains from clade 1. This first *Leucocytozoon* clade has eight haplotypes (lineages) with relatively little variation (uncorrected p = 1.2%). The second Strigidae clade has 28 lineages with an average of 3.7% variation.

**Figure 2:**
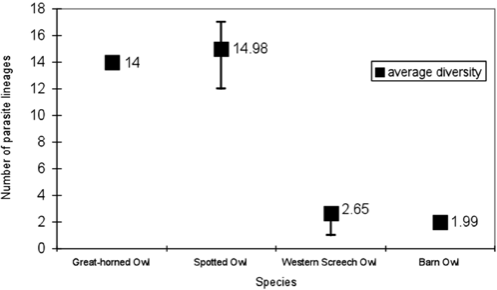
The mean species diversity of Leucocytozoon lineages randomly drawn from four owl species (n = 30).

Host specificity for *Leucocytozoon* spp. appears to differ among owl species. The EcoSim rarefaction species richness test on Barn, Western Screech, Spotted, and Great-horned Owls showed that lineage diversity from 30 randomly selected owls for 1000 replications resulted in a low mean diversity for Western Screech (2.65) and Barn Owls (1.99) and a high mean lineage diversity for Spotted Owls (14.99) and Great-horned (14) (Figure 2). Barn Owls and Western Screech Owls appear to have very specific *Leucocytozoon* strains each with one dominant lineage and one or two less common lineages. In contrast, Spotted Owls and Great-horned Owls showed low specificity and were infected with many different parasite lineages. In addition, we found that Spotted and Great-horned Owls had many unique lineages defined as haplotypes that were not found in any other owl species (Spotted Owls = 12 unique out of 17 total lineages; Great-horned Owls = 10 unique out of 14 total lineages).

#### Plasmodium and Haemoproteus spp

**Figure 3:**
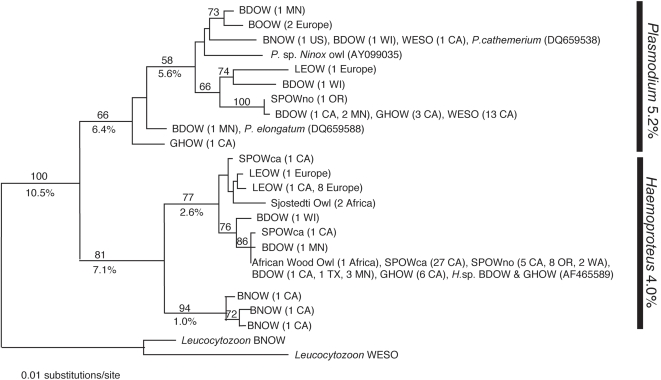
Maximum likelihood tree for *Plasmodium* and *Haemoproteus* parasites in ten owl species. Maximum likelihood bootstrap values are shown above the branches (1000 replicates) and uncorrected ‘p’ distances are shown below. For explanation of bird abbreviation codes see Table 2. Following each bird abbreviation is the number of individuals (n) with that particular lineage and the sampling location.

433 base pairs were sequenced from 106 individuals among 10 owl species. There were 21 unique lineages (10 *Plasmodium* and 11 *Haemoproteus*) found with 139 variable sites and 100 parsimony-informative sites. The maximum likelihood heuristic search yielded one tree for *Plasmodium* and *Haemoproteus* spp. from ten species of owl (Figure 3).

In Figure 3, there appears to be four distinct lineages or possible species of *Plasmodium* present in these owls. One of the *Plasmodium* species from a Barred Owl can be identified as *P. elongatum* because it is an identical match to a sequence identified from GenBank (DQ659588). The other three possible species could only be identified to the genus *Plasmodium* due to the poor quality of available blood smears.

Sequence data identified one Northern Spotted Owl with a *Plasmodium* parasite. The sequence differs by only one base change from a parasite isolated from one invasive Barred Owl but also other native California owls (3 Great-horned Owls, and 13 Western Screech Owls).

In the *Haemoproteus* clade, one dominant lineage (n = 55) was isolated from four owl species (Spotted Owl, Barred Owl, Great-horned Owl, and African Wood Owl) and from North America and Africa. Additionally, Long-eared Owls from North America and Europe shared an identical *Haemoproteus* strain. The global commonality of blood parasites within these owls provides evidence that these blood parasites have no distinct phylogeographic pattern. Also, Barn Owls appear to have a unique *Haemoproteus* lineage although the prevalence of this parasite in the population remains low (1.3%).

## Discussion

### Prevalence of Blood Parasite

The prevalence of blood parasites varied highly among owl species. The lowest prevalences were documented in Barn Owls from Denmark. Four previous studies from northern Europe also detected no blood parasites in Barn Owls (n = 26) [61]–[66]. This suggests that the habitats of Barn Owls in Denmark and other Northern European countries are less suitable for blood parasite vectors [67]. The African owls, in contrast, came from forested habitats with a diverse vector fauna resulting in high prevalences and a high number of co-infections; up to five different haemosporidian species in one individual [68].

The number of Spotted Owls found with a blood parasite (*S . o. caurina* = 52% and *S. o. occidentalis* 79%) in this study, although high, is significantly lower than the 100% prevalence seen in Gutiérrez's (1989) study (p = 0.001; χ^2^ = 59.39). The Northern Spotted Owl prevalence of all haemosporidians over a ten year time span (Table 3) shows that the blood parasite prevalences compared between the years 1994–1996 and 2004–2005 were not statistically different. Therefore, the differences between this study and Gutiérrez's (1989) results may not represent a decline in blood parasite infections over time, but may have been due to other reasons such as differing blood parasite detection methods. This study is the first to use PCR based detection of blood parasites in Spotted Owls, which has been proven to yield much greater sensitivity than older microscopy techniques [69]. Yet, a recent study found although PCR assays were sufficient in finding single parasite infections, it may underestimate cases with multiple parasite infections [46].

Another difference between our study and Gutierrez's is the sampling locations. The 1989 study examined California Spotted Owls from four different locations (Sierra Nevada, San Jacinto Mountains, San Bernadino Mountains, and Palomar Mountains), and the Northern Spotted Owl samples were from northwestern California [19]. The California Spotted Owls in this study came from one Sierra Nevada location, and the Northern Spotted Owls came from California locations as well as Oregon and Washington. Other factors that could contribute to the differing prevalence rates include time of year the owls were sampled, annual variations, age of host [70], and environmental conditions [71].

In a broad sense, the variation in blood parasite prevalences observed between host species and geographic locations in Table 2 could be partially explained by differential exposure to vectors [33], [72]–[74]. Prevalence of a parasite in a host can reflect the amount of contact with a vector; a species with more exposure to vectors should have higher prevalence rates than a species with less exposure [33]. Increased vector exposure leading to increased parasite risks are associated with tropical zones [75], forest habitats [67], migration [73], colonial behavior [76], body size [77], cavity nesting [76], shortened period of embryonic growth [78], and bright plumage coloration [77].

In contrast, research has shown that even taxonomically related bird species sharing similar habitat types may have varying blood parasite prevalences due to differing life histories or vector preferences [72], [73], [76]. For example, black flies and mosquitoes will feed preferentially on certain hosts [79]–[81]. However, one would still anticipate that Spotted Owls and Barred Owls living in sympatry to have comparable infection rates. Our prediction was that these two owl species would have high blood parasite prevalence rates since they share many qualities that would increase vector exposure such as large body size, long life span, cavity nesting, long fledging period, and habitation of forested areas [33], [73], [76]. Spotted and Barred Owls should also have similar prevalences because they are closely-related sister taxa, sharing similar habitats, life histories, relative body size, and plumage coloration [11]. Contradictory to expectations, we found that Barred Owls on the West Coast have significantly lower prevalences for *Leucocytozoon, Haemoproteus,* and fewer cases of simultaneous multiple infections than both subspecies of Spotted Owls. The following three hypotheses can account for differences in Barred Owl and Spotted Owl parasite prevalence: 1) dissimilar micro-habitat preferences leading to differential vector exposure, 2) the West Coast region has a different parasite fauna as compared to the historic Barred Owl range, and in particular fewer *Plasmodium* parasites 3) Barred Owl may have superior immune health. We consider each of these hypotheses below.

First, although Barred Owls and Spotted Owls have overlapping habitat requirements, Barred Owls occupy a wider range of habitats [11]. Barred Owls on the West Coast have been shown to prefer riparian habitats, and unlike Spotted Owls, they can utilize young and mature forests for foraging and breeding [11], [82]. Because of this, it is plausible that their preferred micro-habitats results in differential vector contact, which would cause differing blood parasite prevalences [33].

Secondly, the lower overall prevalence of blood parasites for Barred Owls on the West Coast may be due to less exposure to *Plasmodium*. Another study on blood parasite prevalence did not discover any *Plasmodium* parasites from hawks and owls from the California Raptor Center (n = 55) in Davis, California [83]. We observed that two of 26 Barred Owls (8%) were infected with *Plasmodium* on the West Coast as compared to 6 individuals out of 11 (33%) from the historic range. This suggests that the Barred Owls are susceptible, but show less prevalence of *Plasmodium* spp. in some regions of the western U.S. Perhaps a low abundance or lack of appropriate mosquito vectors in the northern West Coast region could be preventing the spread of *Plasmodium* parasites. This theory would also explain why Spotted Owls have not been documented with *Plasmodium* spp. With a lower risk of *Plasmodium* infections in this area, Barred Owls could have a competitive advantage over Spotted Owls.

Finally, even if *Plasmodium* parasites are rare in the West, Barred Owls should have similar exposure to *Leucocytozoon* and *Haemoproteus*. Instead, the Barred Owls have low parasite prevalences for parasites of all three genera, and the observed discrepancy between Barred and Spotted Owl prevalences could be explained by a better host immune response to the parasites. Furthermore, molecular evidence shows that Northern Spotted Owls have recently experienced a population bottleneck resulting in a loss of genetic variation [84]. This loss of genetic variability may play a role in a reduced ability to cope with blood parasite infections resulting in a weakened immune health.

Symptoms of blood parasite infections can range from mild to severe depending on host susceptibility [85], host fitness status at the time of infection (e.g., nutritional health or reproductive effort) [48], [55], parasite and host genetics [86], acquired immunity [87], and environmental stress [71]. Although this study did not clinically assess symptoms or measure the intensity of parasitemia, we can still assume a higher immune cost associated with multiple parasite infections. In cases of multiple infections, more of the host's resources are being exploited, which increases virulence or severity of the disease [76]. In an extreme example, second year Purple Martins (*Progne subis)* had multiple infections of *Haemoproteus* spp. and a filarial nematode resulted in a 90% fatality rate [88]. The sheer number of Spotted Owls with multiple parasite infections supports the notion that the Spotted Owls have weakened immune systems.

### DNA Sequencing and Host-Parasite Relationships

The *Leucocytozoon* parasites found in eleven owl species studied appear to comprise at least two cryptic species or subspecies. Only one *Leucocytozoon* species (*L. danilewskyi*) has been accepted as valid among all owls, and in this study, *Leucocytozoon* parasites examined microscopically were morphologically indistinguishable (although slide quality was often poor). Yet, there are two distinctly different clades with an average uncorrected *p* sequence divergence of 8.05%. Recent studies of *Haemoproteus* morphospecies suggest that greater than 5% sequence divergence in the cytochrome *b* gene between two lineages is indicative of distinct species [37]. In addition, intraspecific variation is likely to be apparent in *Leucocytozoon* species of owls, as was shown with *L. schoutedeni* of chickens, and in other haemosporidians [34], [38]. Thus, even though *Leucocytozoon danilewskyi* was traditionally thought to be one species, sequencing revealed that it has more than double the number of lineages (n = 36) than *Haemoproteus* (n = 11) and *Plasmodium* (n = 10) parasites in owls. This diversity is probably attributable to a combination of cryptic speciation and intraspecific variation.

Spotted Owls have high lineage diversity with the most *Leucocytozoon* lineages (n = 17) compared to the other owl species, and also 12 lineages that were unique, found only in Spotted Owls hosts. In theory, when a parasite strain has coevolved with a specific host, the parasite imposes a lessened effect on host fitness [89], [90]. Likewise, parasites that are not constrained to a specific host are thought to be more virulent. This has been documented in *Plasmodium* species [43], [44], [91]. The data suggest that *Leucocytozoon* spp. have low host specificity for Spotted Owls, which implies that Spotted Owls have an increased risk of infection by novel parasites with potentially increased virulence.

Although, this is the first study to show a Spotted Owl infected with a *Plasmodium* parasite, there is no conclusive evidence that this parasite originated from Barred Owls since it was also found in other native California owls, and may have already been present at low levels in local bird populations. This study would benefit from larger sample sizes for Barred Owls and Spotted Owls to better determine whether Barred Owls have introduced novel blood parasites. Future work should focus on the combination of blood parasite analysis along with immunity tests and estimated annual survival and reproductive rate for banded Spotted Owls. Utilization of immunity tests and survival estimates would give researchers a better understanding of the effect of blood parasites in the Spotted Owl, and perhaps help determine if some parasite strains are more virulent than others. Additionally, museum specimens of Spotted Owls and other California owls should be tested for blood parasites to see if species of *Plasmodium* have been in this area historically, and to see if Spotted Owls have always had high *Leucocytozoon* lineage diversity.

This study provides a baseline for the distribution of blood parasites and strains in owls. This research suggests that Northern and California Spotted Owls have a fragile immune health due to the high numbers of multiple parasite infections, the possible introduction of *Plasmodium*, and their low parasite specificity. Additionally, Barred Owls have only recently begun to colonize the Sierra Nevada and further research on this population of California Spotted Owls and Mexican Spotted Owls prior to invasion could help reveal the Barred Owl's role in spreading disease and whether or not they contribute to a decline in Spotted Owl immune health. Overall, results are important as conservation measures are planned for all three subspecies of the Spotted Owl.

## Methods

Blood, DNA, and liver tissue samples (n = 542) were donated by eight organizations (Table 4). Blood samples donated from rehabilitation centers were taken from owls when they were first submitted to the clinic. Samples collected from wild birds were taken from apparently healthy individuals that were banded and then released. All blood samples were collected from the years 2003 to 2006, except for some of the Northern Spotted Owls (1 from 1992, 15 from 1994–1995, 5 from 1996, 4 from 2000, 28 from 2004–2005, and 9 unknown dates) and Barred Owls (7 from 1990–1994, 2 from 2001, 22 from 2003–2006, and 13 unknown dates) collected by S. Haig. Most blood parasite analyses were performed on birds sampled during peak transmission times in spring and summer (n = 349 April to September), while 76 were sampled during the autumn and winter months (Oct–Mar), and 117 samples did not have a date of capture.

**Table 4:** Organizations and locations of donated owl tissue samples.

Organization	Sample Location	n	Sample Type
Lindsay Wildlife Museum (Walnut Creek, CA)	USA (CA)	178	blood
School of Veterinary Medicine University of California (Davis, CA)	USA (CA)	83	blood
USDA Forest Service (Davis, CA)	USA (CA)	50	blood
Hungry Owl Project (San Francisco, CA)	USA (CA)	39	blood
California Academy of Science (San Francisco, CA)	USA (CA, OR)	7	liver
United States Geological Survey (Corvallis, OR)	USA (CA, OR, WA, TX, WI)	95	DNA
The Raptor Center (St. Paul, MN)	USA (MN)	8	blood
Zoological Museum at the University of Copenhagen (Denmark)	Denmark	45	blood
Center for Tropical Research (Los Angeles, CA)	Cameroon, Lithuania	35	blood
Russian Academy of Sciences (Rybachy, Russia)	Kazakstan	2	blood

The blood and liver samples (50–100 ul) were stored in lysis buffer (10mM Tris-HCL pH 8.0, 100mM ethylenediaminetetracetic acid, 2% sodium dodecyl sulphate) and kept frozen in a −20°C freezer. However, one set of blood samples from the Zoological Museum University of Copenhagen (n = 45) were not stored in lysis buffer and were kept in −80°C freezers. In some cases, blood smears were also made, fixed in methanol, and stained with Giemsa [92], [93]. Since many blood samples were donated to this study after the bird had been sampled, we do not have blood smears for every sample.

### Blood Parasite Detection

DNA was extracted using a DNeasy kit and following the animal tissue protocol (Qiagen). To test for *Leucocytozoon* spp., extracted DNA was used in a nested PCR reaction that amplifies the cytochrome *b* region of the mtDNA. The first round of amplification used the following primers: DW2: 5′-TAA TGC CTA GAC GTA TTC CTG ATT ATC CAG-3′, and DW4: 5′-TGT TTG CTT GGG AGC TGT AAT CAT AAT GTG-3′ [35]. The first PCR reaction was performed using the following conditions: twenty-five-microliter reaction mixtures contained 10–100 ng of genomic DNA (2 ml of template DNA), 0.5 units of Qiagen Taq DNA Polymerase (Qiagen), 10 mM Tris-HCl (pH 8.3), 50 mM KCl, 3.0 mM MgCl2, 0.4 mM of each primer, 0.4 mM of each dNTP, and 5 ml of Q buffer (Qiagen Inc., Valencia, California). The cycling profile consisted of an initial denaturation at 94°C for 3 min, followed by 35 cycles of 94°C denaturation for 30 sec, 52°C annealing for 30 sec, and 72°C extension for 1 min. The samples went through a final extension at 72°C for 10 min. The second PCR reaction used the first PCR product to seed the reaction instead of DNA template with the following primers: Leuco Cyt F: 5′-TCT TAC TGG TGT ATT ATT AGC AAC-3′, and Leuco Cyt R: 5′-AGC ATA GAA TGT GCA AAT AAA CC-3′ [34]. The reaction conditions using the second primer set was identical to the first round and used a similar cycling profile with a 50°C annealing temperature.

For *Plasmodium* and *Haemoproteus* spp., we used the same PCR reaction conditions as above with the following primers: L15183: 5′-GTG CAA CYG TTA TTA CTA ATT TAT A-3′ and H15730: 5′-CAT CCA ATC CAT AAT AAA GCA T-3′ [94], [95]. The cycling profile consisted of an initial denaturing at 94°C for 3 min, followed by 35 cycles of 94°C for 50 sec, 53°C annealing for 50 sec, and 72°C extension for 60 sec, and then a final extension at 72°C for 5 min.

Positive and negative controls were used for the detection of *Plasmodium*, *Haemoproteus*, and *Leucocytozoon* spp. Positive controls were from birds with known infections evident from microscopy results. Negative controls used purified water in place of DNA template. PCR products were run out on a 1.8% agarose gel using 1xTBE, and visualized by an ethidium bromide stain under ultraviolet light. Some owl samples with good quality blood smears were viewed under a microscope for further parasite detection and to check for accuracy. Slides were examined for 10–15 minutes at low magnification (400×) and then at least 100 fields were studied at high magnification (1000×).

### DNA Sequencing

Bi-directional cycle sequencing was performed using the second primer set (Leuco Cyt F and Leuco Cyt R) for the *Leucocytozoon* spp. samples and the original primer set (L15183 and H15730) for the *Plasmodium* and *Haemoproteus* spp. samples. The positive PCR products were sequenced in an ABI Prism 3100 automated sequencer (Applied Biosystems, Inc., Foster City, CA). For cases where there were multiple parasite infections, evident by double peaks on the chromatogram, the TOPO TA Cloning Kit (Invitrogen Corp., Carlsbad, CA) was used following manufacturer instructions to isolate parasite strains. Ninety seven percent of *Plasmodium* and *Haemoproteus* spp. positives and 79.5% of *Leucocytozoon* spp. positives were successfully sequenced. The remaining 20.5% of *Leucocytozoon* positives could not be sequenced due to illegible sequence profiles, unsuccessful cloning, or insufficient quantities of DNA template.

The sequences were deposited in GenBank with the following accession numbers EU627791-EU627845.

### Analysis

All DNA sequences were edited using Sequencher 3.1 (GeneCodes, Ann Arbor, MI) and MacClade 3.0 and 4.03 PPC [96]. For distinguishing between *Plasmodium* and *Haemoproteus* spp., the sequences were compared to their closest sequence matches in GenBank using the NCBI nucleotide blast search and by confirmation through microscopy slides. Modeltest 3.7 [97] was used to identify the best model for each dataset; GTR+G was chosen for *Leucocytozoon, Plasmodium,* and *Haemoproteus* spp.

Phylogenetic relationships were constructed for *Leucocytozoon* and *Plasmodium* and *Haemoproteus* spp. parasites in PAUP* 4.0b10 [98]. A maximum likelihood heuristic search with 100 replicates for *Leucocytozoon* and 1000 replicates *Plasmodium* and *Haemoproteus* spp was performed with a TBR branch-swapping algorithm and a neighbor-joining tree used as the starting tree.

In order to summarize the results, the bootstrap values and genetic distances, estimated using the uncorrected ‘p’ distance setting, were added to the final maximum likelihood phylograms. Parsimony and neighbor-joining trees were computed for all parasites and all showed similar topographies as the maximum likelihood trees.

A *Plasmodium* parasite from an owl and *Leucocytozoon toddi* were chosen as the outgroups for the *Leucocytozoon* trees, and an owl *Leucocytozoon* was the designated outgroup for the *Plasmodium/Haemoproteus* tree. These outgroups were chosen because *Plasmodium* and *Leucocytozoon* are sister taxa [35]. A *Leucocytozoon toddi* sequence (DQ177250) from GenBank was also placed in the *Leucocytozoon* phylogram to further resolve the backbone of the tree. Three additional sequences for the *Plasmodium/Haemoproteus* analyses were obtained from GenBank including: *Haemoproteus* sp. from a Great-horned Owl and Barred Owl (AF465589), *Plasmodium* sp. from a Singapore Brown Hawk Owl (*Ninox scutulata*) (AY099035), and *Plasmodium elongatum* from a Great Blue Heron (*Ardea herodias*) (DQ659588).

Statistical comparisons of parasite prevalence among owl species were conducted as binomial comparative trials with results presented as Yates corrected Chi Square's. A *p* value of 0.05 or less was considered significant. We also tested for *Leucocytozoon* lineage diversity among the four owl species with largest sample sizes (Barn Owl, Western Screech Owl, Spotted Owl, and Great-horned Owl) using EcoSim 7.0 that provides rarefaction estimates [99]. The program randomly sampled 25 lineages from each owl species for 1000 iterations to create a mean and variance of lineage diversity.

## References

[1] Daszak P, Cunningham AA, Hyatt AD (2000). Emerging infectious diseases of wildlife-threats to biodiversity and human health.. Science.

[2] Harvell CD, Mitchell CE, Ward JR, Altizer S, Dobson AP (2002). Climate warming and disease risks for terrestrial and marine biota.. Science.

[3] Shea K, Thrall PH, Burdon JJ (2000). An integrated approach to management in epidemiology and pest control.. Ecol Lett.

[4] Woodworth BL, Atkinson CT, LaPointe DA, Hart PJ, Spiegal CS (2005). Host population persistance in the face of introduced vector-borne diseases: Hawaii amakihi and avian malaria.. P Natl Acad Sci USA.

[5] Rappole JH, Hubálek Z (2006). Birds and influenza H5N1 virus movement to and within North America.. Emerg Infect Dis.

[6] Komar N, Langevin S, Hinten S, Nemeth N, Edwards E (2003). Experimental infection of North American birds with the New York 1999 strain of West Nile Virus.. Emerg Infect Dis.

[7] Fauci AS (2003). HIV and AIDS: 20 years of science.. Nat Med.

[8] Daszak P, Cunningham AA (2003). Anthropogenic change, biodiversity loss, and a new agenda for emerging diseases.. J Parasitol.

[9] Kilpatrick AM, LaPointe DA, Atkinson CT, Woodworth BL, Lease JK (2006). Effects of chronic avian malaria (Plasmodium relictum) infection on reproductive success of Hawaii Amakihi (Hemignathus virens).. Auk.

[10] Deredec A, Courcham F (2001). Sex-specific associations between reproduction output and hematozoan parasites of American Kestrels.. Oecologia.

[11] Gutiérrez RJ, Cody M, Courtney S, Kennedy D, Courtney SP, Blakesley J, Bigley R, Cody M, Dumbacher J (2004). Assessment of the potential threat of the Northern Barred Owl.. Scientific evaluation of the status of the Northern Spotted Owl.

[12] Sakai H (2005). Range expansion of the Barred Owls into Redwood National and State Parks: management implications and consequences for threatened Northern Spotted Owls.. Park Science.

[13] US Department of the Interior (1990). U.S. Fish and Wildlife Service. Endangered and Threatened Wildlife and Plants: Determination of Threatened Status for the Northern Spotted Owls Final Rule.. Federal Register, Part VI.

[14] Dark SJ, Gutierrez RJ, Gould GI (1998). The invasion of Barred Owls (Strix varia) in California: Potential impacts on the Spotted Owl (Strix occidentalis).. Auk.

[15] Courtney SP, Franklin A, Courtney SP, Blakesley JA, Bigley RE, Cody ML, Dumbacher J (2004). Information Needs.. Scientific evaluation of the status of the Northern Spotted Owl.

[16] Grant J (1966). The Barred Owl in British Columbia.. Murrelet.

[17] Hume M (2007). B.C.'s spotted owl near extinction. The Globe and Mail. Toronto.

[18] Taylor AL, Forsman ED (1976). Recent range extensions of the Barred Owl in Western North American including the first records for Oregon.. Condor.

[19] Monahan WB, Hijmans RJ (2007). Distributional dynamics of invasion and hybridization by Strix spp. in Western North America.. Ornithol Monogr.

[20] Gutiérrez RJ, Franklin AB, Lahaye WS, Poole A, Gill F (1995). Spotted Owl (Strix occidentalis).. The American Ornithologists' Union.

[21] Gutiérrez RJ (1989). Hematozoa from the Spotted Owl.. J Wildlife Dis.

[22] La Haye W, Courtney SP, Blakesley JA, Bigley RE, Cody ML, Dumbacher JP (2004). Northern Spotted Owl Biology.. Scientific evaluation of the status of the Northern Spotted Owl.

[23] Noon BR, Franklin AB (2002). Scientific research and the Spotted Owl (Strix occidentalis): opportunities for major contributions to avian population ecology.. Auk.

[24] Greiner EC, Bennett GF, White EM, Coombs RF (1975). Distribution of the avian hematozoa of North America.. Can J Zool.

[25] Telford SR, Forrester DJ (1992). Morphological comparisons of the Plasmodium (Novyella) species reported from North American birds, with comments on a species from the barred owl Strix varia Barton.. Syst Parasitol.

[26] Williams NA, Bennett GF (1978). Hematozoa of some birds of New Jersey and Maryland.. Can J Zool.

[27] Wetmore PW (1941). Blood parasites of the District of Columbia and Patuxent Research Refuge vicinity.. J Parasitol.

[28] Telford SR, Nayar JK, Foster GW, Knight JW (1997). Plasmodium forresteri n.sp., from raptors in Florida and southern Georgia: Its distinction from Plasmodium elongatum morphologically within and among host species and by vector susceptibility.. J Parasitol.

[29] Olsen GH, Gaunt SD (1985). Effect of hemoprotozoal infections on rehabilitation of wild raptors.. J Am Vet Med Assoc.

[30] Hart JW (1949). Observations on blood parasites of birds in South Carolina.. J Parasitol.

[31] Forrester DJ, Telford SR, Foster GW, Bennett GF (1994). Blood parasites of raptors in Florida.. J Raptor Res.

[32] Kirkpatrick CE, Lauer DM (1985). Hematozoa of raptors from southern New Jersey and adjacent areas.. J Wildlife Dis.

[33] Valkiunas G (2005). Avian malaria parasites and other Haemosporidia..

[34] Sehgal RNM, Hull AC, Anderson NL, Valkiunas G, Markovets MJ (2006). Evidence for Cryptic Speciation of Leucocytozoon spp. (Haemosporida, Leucocytozoidae) in Diurnal Raptors.. J Parasitol.

[35] Perkins SL, Schall JJ (2002). A molecular phylogeny of malarial parasites recovered from cytochrome b gene sequences.. J Parasitol.

[36] Bensch S, Pérez-Tris J, Waldenstrom J, Hellgren O (2004). Linkage between nuclear and mitochondrial DNA sequences in avian malaria parasites: multiple cases of cryptic speciation?. Evolution.

[37] Hellgren O, Križanuskiene A, Valkiunas G, Bensch S (2007). Diversity and phylogeny of mitochondrial cytochrome b lineages from six morphospecies of avian Haemoproteus (Haemosporidia: Haemoproteidae).. J Parasitol.

[38] Palinauskas V, Kosarev V, Shapoval A, Bensch S, Valkiunas G (2007). Comparison of mitochondrial cytochrome b lineages and morphospecies of two avian malaria parasites of the subgenera Haemamoeba and Giovannolaia (Haemosporidia: Plasmodiidae).. Zootaxa.

[39] Ricklefs RE, Fallon SM, Bermingham E (2004). Evolutionary relationships, cospeciation, and host switching in avian malaria parasites.. Syst Biol.

[40] Beadell JS, Ishtiaq F, Covas R, Melo M, Warren BH (2006). Global phylogeographic limits of Hawaii's avian malaria.. P Roy Soc Lond B.

[41] Hellgren O, Waldenstrom J, Perez-Tris J, Szollosi E, Hasselquist D (2006). Detecting shifts of transmission areas in avian blood parasites- a phylogenetic approach.. Mol Ecol.

[42] Miltgen F, Landau I, Ratanaworabhan N, Yenbutra S (1981). Parahaemoproteus desseri n. sp.; Gamétogonie et schizogonie chez l'hôte naturel: Psittacula roseata de Thailande, et sporogonie expérimentale chez Culicoides nubeculosus.. Annales de Parasitologie Humaine et Comparee.

[43] Atkinson CT, Woods KL, Dusek RJ, Sileo LS, Iko WM (1995). Wildlife disease and conservation in Hawaii: pathogenicity of avian malaria (Plasmodium relictum) in experimentally infected Iiwi (Vestiaria coccinea).. Parasitol.

[44] Atkinson CT, Dusek RJ, Woods KL, Iko WM (2000). Pathogenicity of avian malaria in experimentally-infected Hawaii Amakihi.. J Wildlife Dis.

[45] Remple JD (2004). Intracellular Hematozoa of raptors: a review and update.. J Avian Med Surg.

[46] Valkiunas G, Bensch S, Iezhova TA, Križanauskiene A, Hellgren O (2006). Nested cytochrome b polymerase chain reaction diagnostics underestimate mixed infections of avian blood Haemosporidian parasites: microscopy is still essential.. J Parasitol.

[47] Marzal A, De Lope F, Navarro C, Moller AP (2005). Malaria parasites decrease reproductive success: an experimental study in a passerine bird.. Oecologia.

[48] Nordling D, Anderson M, Zohari S, Gustafsson L (1998). Reproductive effort reduces specific immune response and parasite resistance.. P Roy Soc Lond B.

[49] Leppert LL, Layman S, Bragin EA, Katzner T (2004). Survey for Hemoparasites in Imperial Eagles (Aquila heliaca), Steppe Eagles (Aquila nipalensis), and White-tailed Sea Eagles (Haliaeetus albicilla) from Kazakhstan.. J Wildlife Dis.

[50] Hunter BD, Rohner C, Currie DC (1997). Mortality in fledgling Great-horned Owls from black fly Hematophaga and Leucocytozoonosis.. J Wildlife Dis.

[51] Clarabuch O, Gonzalez-Solis J (1998). Parasitism as a migration cost.. Biologia e Conservazione della Fauna.

[52] Dawson RD, Bortolotti GR (2000). Effects of hematozoan parasites on condition and return rates of American Kestrels.. Auk.

[53] Stjernman M, Raberg L, Nilsson JA (2004). Survival costs of reproduction in the blue tit (Parus caeruleus): a role for blood parasites?. Proc R Soc Lond B.

[54] Korpimaki E, Hakkarainen H, Bennett GF (1993). Blood parasites and reproductive success of Tengmalm's owls: detrimental effects on females but not on males?. Functional Ecology.

[55] Appleby BM, Anwar MA, Petty SJ (1999). Short-term and long-term effects of food supply on parasite burdens in Tawny owls, Strix aluco.. Funct Ecol.

[56] Andrezj D (2005). Male reproductive success is correlated with blood parasite levels and body condition in the promiscuous aquatic warbler (Acrocephalus paludicola).. Auk.

[57] Dulfa R (1996). Blood parasites, health, reproductive success, and egg volume in female Great Tits Parus major.. J Avian Biol.

[58] Korpimaki E, Tolonen P, Bennett GF (1995). Blood parasites, sexual selection and reproductive success of European kestrels.. Ecoscience.

[59] Hakkarainen H, Ilmonen P, Koivunen V, Korpimäki E (1998). Blood parasites and nest defense behavior of Tengmalm's owls.. Oecologia.

[60] Sanz JJ, Arriero E, Moreno J, Merino S (2001). Female Hematozoan infection reduces hatching success but not fledging success in Pied Flycatchers Ficedula hypoleuca.. Auk.

[61] Peirce MA, Greenwood AG, Cooper JE (1983). Haematozoa of raptors and other birds from Britian, Spain, and the United Arab Emirates.. Avian Pathol.

[62] Peirce MA, Marquiss M (1983). Haematozoa of British birds. VII. Haematozoa of raptors in Scotland with a description of Haemoproteus nisi sp. nov. from the sparrow-hawk (Accipiter nisus).. J Nat Hist.

[63] Sacchi L, Prigioni C (1984). Occurance of Leucocytozoon and Haemoproteus (Apicomplexa, Haemosporina) in Falconiformes and Strigiformes of Italy.. Annales de Parasitologie Humaine et Comparee.

[64] Mikaelian I, Bayol P (1991). Blood protozoa found in birds of prey undergoing rehabilitation.. Le Point Veterinare.

[65] Lierz M, Gobel T, Schuster R (2002). Occurrence of parasites in indigenous birds of prey and owls.. Berliner und Munchener Tierartztiche Wochenschrift.

[66] Krone O, Priemer J, Streich J, Sommer P, Langgemach T (2001). Haemosporida of birds of prey and owls from Germany.. Acta Protozoologica.

[67] Tella JL, Blanco G, Forero MG, Gajón Á, Donázar JA (1999). Habitat, world geographic range, and embryonic development of hosts explain the prevalence of avian hematozoa at a small spatial and phylogenetic scales.. P Natl Acad Sci USA.

[68] Sehgal RNM, Jones HI, Smith TB (2005). Blood Parasites of Some West African Rainforest Birds.. J Vet Med Sci.

[69] Richard FA, Sehgal RNM, Jones HI, Smith TB (2002). A comparative analysis of PCR-based detection methods for avian malaria.. J Parasitol.

[70] Deviche P, Greiner EC, Manteca X (2001). Seasonal and age-related changes in blood parasites prevalence in dark-eyed juncos (Junco hyemalis, Aves, Passeriformes).. J Exp Zool.

[71] Oppliger A, Clobert J, Lecomte J, Lorenzon P, Boudjemadi K (1998). Environmental stress increases the prevalence and intensity of blood parasite infection in the common lizard Lacerta vivipara.. Ecol Lett.

[72] Yezerinac SM, Weatherhead PJ (1995). Plumage coloration, differential attraction of vectors and haematozoa infections in birds.. J Anim Ecol.

[73] Møller AP, Erritozøe J (1998). Host immune defence and migration in birds.. Evol Ecol.

[74] Møller AP, Martin-Vivaldi M, Soler JJ (2004). Parasitism, host immune defence and dispersal.. J Evol Biol.

[75] Durrant KL, Beadell JS, Ishtiaq F, Graves GR, Olson SL (2006). Avian hematozoa in South America: a comparison of temperate and tropical zones.. Ornithol Monogr.

[76] Merino S, Møller AP, Adams NJ, Slotow RH (1999). The coevolution of virulence and immune defence in birds.. Proc 22 Int Ornithol Congr, Durban.

[77] Scheuerlein A, Ricklefs RE (2004). Prevalence of blood parasites in European passeriform birds.. P Roy Soc Lond B.

[78] Ricklefs RE (1992). Embryonic development period and the prevalence of avian blood parasites.. P Roy Soc Lond B.

[79] Molaei G, Andreadis TG, Armstrong PM, Anderson JF, Vossbrinck CR (2006). Host feeding patterns of Culex mosquitoes and West Nile virus transmission, northeastern United States.. Emerg Infect Dis.

[80] Malmquist B, Strasevicius D, Hellgren O, Adler PH, Bensch S (2004). Vertebrate host specificity of wild-caught blackflies revealed by mitochondrial DNA in blood.. P Roy Soc Lond B.

[81] Kilpatrick AM, Kramer LD, Jones MJ, Marra PP, Daszak P (2006). West Nile Virus epidemics in North America are driven by shifts in mosquito feeding behavior.. PLoS Biol.

[82] Levy S (1999). Owl vs. Owl.. J Nat Hist.

[83] Ziman M, Colagross-Schouten A, Griffey S, Stedman B (2004). Haemoproteus spp. and Leukocytozoon spp. in a Captive Raptor Population.. J Wildlife Dis.

[84] Funk CW, Forsman ED, Mullins TD, Haig SM (2008). Pleistocene expansion followed by recent population bottlenecks in northern spotted owls.. P Roy Soc Lond B: submitted.

[85] Atkinson CT, Dusek RJ, Lease JK (2001). Serological responses and immunity to superinfection with avian malaria in experimentally infected Hawaii Amakihi.. J Wildlife Dis.

[86] Mackinnon MJ, Gunawardena DM, Rajakaruna J, Weerasingha S, Mendis KN (2000). Quantifying genetic and nongenetic contributions to malarial infection in a Sri Lanka population.. P Natl Acad Sci USA.

[87] Gatton ML, Cheng Q (2004). Modeling the development of acquired clinical immunity to Plasmodium falciparum malaria.. Infect Immun.

[88] Davidar P, Morton ES (2006). Are multiple infections more severe for Purple Martins (Progne subis) than single infections?. Auk.

[89] Beadell JS, Gering E, Austin J, Dumbacher JP, Pierce MA (2004). Prevalence and differential host-specificity of two avian blood parasite genera in the Australo-Papuan region.. Mol Ecol.

[90] Bush AO, Fernandez JC, Esch GW, Seed JR, Press CU (2001). Parasitism: The diversity and ecology of animal parasites;.

[91] Bensch S, Stjernman M, Hasselquist D, Ostman O, Hansson B (2000). Host specificity in avian blood parasites: a study of Plasmodium and Haemoproteus mitochondrial DNA amplified from birds.. P Roy Soc Lond B.

[92] Godfrey RD, Fedynich AM, Pence DB (1987). Quantification of hematozoa in blood smears.. J Wildlife Dis.

[93] Valkiunas G, Iezhova TA (1997). A comparison of the blood parasites in three subspecies of the Yellow Wagtail Motacilla flava.. J Parasitol.

[94] Szymanski MM, Lovette IJ (2005). High lineage diversity and host sharing of malarial parasites in a local avian assemblage.. J Parasitol.

[95] Fallon SM, Bermingham E, Ricklefs RE (2003). Island and taxon effects in parasitism revisited: avian malaria in the Lesser Antilles.. Evolution.

[96] Maddison D, Maddison W (2002). MacClade 4.03 PPC : analysis of phylogeny and character evolution. 4.03 PPC ed..

[97] Posada D, Crandall KA (1998). Modeltest: testing the model of DNA substitution.. Bioinformatics.

[98] Swofford DL (2002). PAUP* Phylogenetic Analysis Using Parsimony (*and other methods) version 4.0b10..

[99] Gotelli NJ, Entsminger GL (2006). EcoSim: Null models software for ecology. 7 ed.. http://garyentsminger.com/ecosim.htm.

